# Insights Into the Mechanisms of Biological Aging That Guide Translational and Clinical Research

**DOI:** 10.1002/agm2.70107

**Published:** 2026-09-05

**Authors:** Evandro Fei Fang, Hilde Nilsen, Morten Scheibye‐Knudsen, Vilhelm A. Bohr, Lin Kang

**Affiliations:** ^1^ Department of Clinical Molecular Biology University of Oslo and Akershus University Hospital Lørenskog Norway; ^2^ The Norwegian Centre on Healthy Ageing (NO‐Age) and the Norwegian National Anti‐Alzheimer's Disease (NO‐AD) Networks Oslo Norway; ^3^ Institute of Clinical Medicine, University of Oslo Oslo Norway; ^4^ Centre for Embryology and Healthy Development (CRESCO) University of Oslo Oslo Norway; ^5^ Department of Microbiology Oslo University Hospital Oslo Norway; ^6^ Center for Healthy Aging, Department of Cellular and Molecular Medicine University of Copenhagen Copenhagen Denmark; ^7^ Section on DNA Repair National Institute on Aging Baltimore Maryland USA; ^8^ Department of Geriatrics Peking Union Medical College, Chinese Academy of Medical Sciences, Peking Union Medical College Hospital Beijing China

**Keywords:** aging, autophagy, brain, DNA repair, mitophagy, NAD^+^

## Abstract

Understanding the molecular mechanisms of aging guides the development of prevention, intervention, and treatment strategies to reduce the incidence of common age‐associated diseases. Over the past decades, the proposed key features (hallmarks) of aging cells have directly or indirectly provided us clues, furthering our understanding of the causes of disease and assisting with the development of therapeutic strategies for diseases such as rare premature aging diseases like Werner syndrome and ataxia telangiectasia (A‐T), as well as the common age‐related diseases like dementia and sarcopenia. In this editorial, we take a closer look at three of these hallmarks including genomic instability, defective macroautophagy, and mitochondrial dysfunction, and the applications of these concepts in understanding the progress of complex diseases. Mounting studies from the laboratory, supported by emerging clinical evidence, point to the reduction of the oxidized form of nicotinamide adenine dinucleotide (NAD^+^) as a commonality between many of these hallmarks of aging. Intriguingly, stimulating mitochondrial autophagy (mitophagy) via improvement of NAD^+^ availability appears to be a promising and effective therapeutic strategy for many diseases relating to aging. Future studies on the hallmarks of aging should address their internal linkages and clinical interventions.

## Graphical abstract

Cellular NAD+ levels can be augmented through supplementation with precursors such as nicotinamide (NAM), nicotinamide riboside (NR), and nicotinamide mononucleotide (NMN). Accumulating preclinical and clinical evidence suggests that NAD+ repletion attenuates both pathological (premature or accelerated) and chronological aging, thereby extending healthspan. Mechanistically, NAD+ counteracts several hallmarks of aging, including DNA damage accumulation, impaired autophagy/mitophagy, and mitochondrial dysfunction. We propose a NAD^+^‐mitophagy axis which safeguards brain resilience.

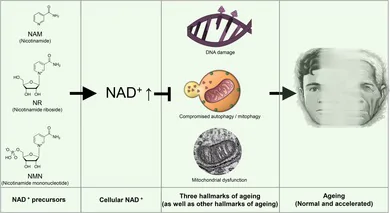

We are now living in an aging society which is associated with formidable healthcare and socioeconomic pressures [1, 2, 3]. A society with an “aging population” can be defined as one where more than 7% of people are over 65 years old, while the term “aged population” is used when 14% or more are over 65 [4]. Age is the primary driver of many diseases, such as cancer, cardiovascular disease, sarcopenia, and neurodegenerative diseases like Alzheimer's disease (AD) and Parkinson's disease (PD). One of the major discoveries in aging has been that the rate of aging is driven, at least to some extent, by genetic pathways and biochemical processes conserved in evolution [5]. Scientists, especially molecular gerontologists (scientists who work on aging at the molecular level), have proposed that slowing down aging may delay the onset and the progression of many diseases. Therefore, targeting cellular aging hallmarks is likely to give a cost‐effective strategy for achieving healthy longevity [6].

To guide the development of strategies to reduce the pace of aging, we need to understand the causes of aging, both external (e.g., exposures) and internal factors and their interaction. In 2013, nine hallmarks of aging were proposed, including genomic instability, telomere attrition, epigenetic alterations, loss of proteostasis (including compromised autophagy), deregulated nutrient sensing, mitochondrial dysfunction, altered intercellular (as well as organ‐to‐organ) communication, cellular senescence, and stem cell exhaustion [5]. Since then, we have argued that the scientific evidence points to compromised autophagy being its own independent hallmark of aging. Although there are overlaps between loss of proteostasis and compromised autophagy, autophagy has many unique functions (e.g., degradation and maintenance of many nonprotein targets, such as DNA, RNA, lipid, nuclear structures, mitochondria, and even infected viruses) that distinguish it as an independent hallmarks of aging (as detailed elsewhere [7, 8]). At the 2022 New Hallmarks of Aging symposium in Copenhagen, in the meeting celebrating the lifelong contribution of Dr. Vilhelm A. Bohr to the molecular mechanisms and intervention of aging, at least 5 new hallmarks of aging were proposed: compromised autophagy, microbiome disturbance, altered mechanical properties, splicing dysregulation, inflammation, and “x” (representing still unknown hallmarks) [9]. In 2023, the 2nd version of the consensus hallmarks of aging added three new hallmarks including disabled macroautophagy, chronic inflammation, and dysbiosis (an imbalance in the microbiota within the body, often the gut), bringing the total number of recognized hallmarks to twelve [10].

New data suggest that it is not only “disabled macroautophagy” (one of the three types of autophagy), but “disabled autophagy” as a pathway is a hallmark of aging. In autophagy, the main intracellular “garbage” clearance system, the cell uses a variety of different routes to deliver cellular debris (including partial or whole organelles) to acidic lysosomes for degradation and recycling [11]. There are three types of autophagy: macroautophagy (the most studied), chaperone‐mediated autophagy, and microautophagy; compromises in these pathways are known to contribute to physiological aging, accelerated aging, and age‐predisposed diseases [8]. While there are many reports on the role of disabled macroautophagy in driving aging, recent studies also showed a role for age‐dependent impairment of chaperone‐mediated autophagy; stimulation of chaperone‐mediated autophagy has been shown to slow aging in mice [12, 13]. Additionally, microautophagy also prevents aging [14, 15]. Thus, further studies looking at the role of dysfunction in the various types of autophagy pathways during healthy aging are needed, especially in humans.

The application of the hallmarks of aging has advanced our understanding of the etiology of diseases, especially those with complex physiological mechanisms. Over the past 13 years, we (Fang, Nilsen, Scheibye‐Knudsen, and Bohr), along with our colleagues, have focused extensively on the contribution of at least three hallmarks of aging (genomic instability, compromised autophagy (especially its subtype compromised mitophagy), and mitochondrial dysfunction), focusing on their contribution to premature aging diseases such as Werner syndrome, A‐T, Cockayne syndrome, and xeroderma pigmentosum [16, 17, 18], and have applied our discoveries to advance our understanding of AD [16, 17] and PD [19, 20]. We show a nuclear DNA damage signaling to mitochondria in driving aging due to impairment of the NAD^+^/SIRT1‐mitophagy axis [16, 17]. Encouragingly, our laboratory discoveries related to dysfunctional autophagy, especially through the NAD^+^/SIRT1‐mitophagy axis, have been successfully translated to clinic including for the treatment of A‐T [21] and Werner [22] syndrome using a simple and seemingly safe NAD^+^ augmentation strategy. In AD, we have shown that the compromised NAD^+^/SIRT1‐mitophagy axis is a key mechanism of aging that predisposes to AD [23]; clinical trials in humans are now testing various treatment strategies including the use of NAD^+^ augmentation (see a recent review summary [24]) and the treatment potential of the mitophagy inducing molecule urolithin A (one co‐led by Fang and Vyhnálek [25] while the other by Bohr and Treebak).

In addition to premature aging and AD, stimulating the NAD^+^‐driven mitophagy axis may also slow down sarcopenia, a key feature of frailty in aging. For instance, a newly proposed NAD^+^ precursor, trigonelline, was found to be reduced in human sarcopenia and supplementation of aged mice with this compound improved muscle function [26]. Further, a phase II clinical trial testing supplementation with urolithin A showed improved muscle strength and exercise performance in middle‐aged adults when compared with treatment with placebo [27].

Further clinical studies to validate the efficacy of stimulating the NAD^+^/SIRT1‐mitophagy axis for treating premature aging diseases, AD, sarcopenia, and other diseases are necessary. The new field named geroscience, which aims to identify actionable hallmarks of aging [28], continues to provide exciting new scientific breakthroughs such as newly proposed human aging biomarkers [29, 30] and aging clocks for various physiological systems [31]. Additionally, combinational effects between NAD^+^ and other approaches, such as dietary restriction [32] and new mitophagy inducers [33], are needed. As research into the molecular drivers behind the aging process continues, benefits related to novel scientific and technological advances for early disease diagnosis and intervention will be made available, bringing the prospects that more people will live the latter years in better and healthier ways.

## Author Contributions

E.F.F.: conceptualization and drafted the manuscript and the figure. H.N.: revised the manuscript. M.S.‐K: revised the manuscript. V.A.B.: revised the manuscript. L.K.: coordination, revised the manuscript.

## Funding

E.F.F. is supported by Cure Alzheimer's Fund (#282952; #284930), HELSE SØR‐ØST (#2020001, #2021021, #2023093), the Research Council of Norway (#262175, #334361), Molecule AG/VitaDAO (#282942), NordForsk Foundation (#119986). Lin Kang is supported by National Outstanding Young Physicians under the National High‐Level Medical Talent program and 2025 Standardized Construction Project of Geriatrics Department in Beijing, Beijing Municipal Health Commission.

## Conflicts of Interest

E.F.F. is a co‐owner of Fang‐S Consultation AS (Organization number 931410717) and NO‐Age AS (Organization number 933219127); he has an MTA with LMITO Therapeutics Inc. (South Korea), a CRADA arrangement with ChromaDex (USA), a commercialization agreement with Molecule AG/VITADAO, an MTAs with GeneHarbor (Hong Kong) Biotechnologies Limited, and a data license option agreement with Hong Kong Longevity Science Laboratory (Hong Kong); he is a consultant to MindRank AI (China), NYO3 (Norway), AgeLab (Vitality Nordic AS, Norway), and Hong Kong Longevity Science Laboratory (Hong Kong). V.A.B. is an advisor for Niagen Bioscience. H.N. is a co‐owner of NO‐Age AS (Organization number 933219127), has a CRADA arrangement with Niagen Biosceince, and is an advisor for AgeLab (Vitality Nordic AS, Norway).

## Data Availability

Data sharing not applicable to this article as no datasets were generated or analysed during the current study.
